# Associations between Liver Enzymes, Lifestyle Risk Factors and Pre-Existing Medical Conditions in a Population-Based Cross-Sectional Sample

**DOI:** 10.3390/jcm12134276

**Published:** 2023-06-26

**Authors:** Onni Niemelä, Aini Bloigu, Risto Bloigu, Mauri Aalto, Tiina Laatikainen

**Affiliations:** 1 Department of Laboratory Medicine and Medical Research Unit, Seinäjoki Central Hospital and Tampere University, 60220 Seinäjoki, Finland; 2 Research Unit of Population Health, Faculty of Medicine, University of Oulu, 90014 Oulu, Finland; abloigu@outlook.com; 3 Infrastructure for Population Studies, Faculty of Medicine, University of Oulu, 90014 Oulu, Finland; rbloigu@gmail.com; 4 Department of Psychiatry, Seinäjoki Central Hospital and Tampere University, 33100 Tampere, Finland; mauri.aalto@tuni.fi; 5 Department of Public Health and Social Welfare, Finnish Institute for Health and Welfare (THL), 00271 Helsinki, Finland; tiina.laatikainen@thl.fi; 6 Institute of Public Health and Clinical Nutrition, University of Eastern Finland, 70211 Kuopio, Finland; 7 Joint Municipal Authority for North Karelia Social and Health Services, 80210 Joensuu, Finland

**Keywords:** biomarker, ethanol, hypertension, liver, obesity, physical activity

## Abstract

While alanine aminotransferase (ALT) and gamma-glutamyltransferase (GGT) enzymes are commonly used indicators of liver dysfunction recent studies have suggested that these may also serve as predictive biomarkers in the assessment of extrahepatic morbidity. In order to shed further light on the interactions between serum liver enzyme abnormalities, factors of lifestyle and health status we examined ALT and GGT activities in a population-based sample of 8743 adult individuals (4048 men, 4695 women from the National FINRISK 2002 Study, mean age 48.1 ± 13.1 years) with different levels of alcohol drinking, smoking, physical activity, body weight and the presence or absence of various pre-existing medical conditions. The assessments also included laboratory tests for inflammation, lipid status and fatty liver index (FLI), a proxy for fatty liver. The prevalence of ALT and GGT abnormalities were significantly influenced by alcohol use (ALT: *p* < 0.0005 for men; GGT: *p* < 0.0005 for both genders), smoking (GGT: *p* < 0.0005 for men, *p* = 0.002 for women), adiposity (*p* < 0.0005 for all comparisons), physical inactivity (GGT: *p* < 0.0005; ALT: *p* < 0.0005 for men, *p* < 0.05 for women) and coffee consumption (*p* < 0.0005 for GGT in both genders; *p* < 0.001 for ALT in men). The total sum of lifestyle risk factor scores (LRFS) influenced the occurrence of liver enzyme abnormalities in a rather linear manner. Significantly higher LRFS were observed in the subgroups of individuals with pre-existing medical conditions when compared with those having no morbidities (*p* < 0.0005). In logistic regression analyses adjusted for the lifestyle factors, both ALT and GGT associated significantly with fatty liver, diabetes and hypertension. GGT levels also associated with coronary heart disease, angina pectoris, cardiac insufficiency, cerebrovascular disease, asthma and depression. Combinations of abnormal ALT and GGT activities significantly increased the odds for hypertension coinciding with abnormalities in biomarkers of inflammation, lipid status and FLI. The data indicates that ALT and GGT activities readily respond to unfavorable factors of lifestyle associating also with a wide array of pre-existing medical conditions. The data supports close links between both hepatic and extrahepatic morbidities and lifestyle risk factors and may open new insights on a more comprehensive use of liver enzymes in predictive algorithms for assessing mechanistically anchored disease conditions.

## 1. Introduction

Common laboratory tests for liver dysfunction, alanine aminotransferase (ALT) and gamma-glutamyltransferase (GGT) show important physiological functions in chemical reactions in the body, including breakdown of food into energy (ALT), metabolism of drugs and toxins and adaptation to oxidative stress (GGT). Recent studies have suggested that increases in the activities of these enzymes may also yield predictive value in the assessment of extrahepatic conditions, such as cardiovascular diseases [1,2,3,4,5,6].

Accumulating evidence has recently emphasized the role of modifiable risk factors of lifestyle as determinants of individual health [7]. Excessive alcohol drinking has long been recognized as a major contributor to a large number of particular diseases [8,9]. The metabolic consequences of alcohol drinking as well as other unfavorable lifestyle factors, such as smoking, excess body weight and lack of physical activity, may work individually or in concert to create adverse health effects in a supra-additive manner [7,10,11,12,13,14,15]. Previous studies on the impact of unfavorable lifestyle factors on health have also indicated that the early changes in liver function, the status of inflammation and oxidative stress in the sequence of events leading from risk factor exposure to tissue damage may be reflected in common laboratory tests sensitive to such metabolic aberrations [13]. 

As of yet, the factors underlying the early changes in serum liver enzyme activities and the medical significance of such aberrations in individuals with multiple health risk factors have, however, remained poorly defined. In this work we sought to examine the associations between ALT and GGT abnormalities and various lifestyle risk factors in individuals with and without distinct pre-existing medical conditions in a large national population-based health survey (FINRISK). The study participants were classified according to alcohol drinking, smoking, physical activity, coffee consumption and anthropometric measures. For comparisons, assays of CRP (a biomarker of inflammation), lipid status (cholesterol, HDL-cholesterol, LDL-cholesterol, triglycerides) and fatty liver index (FLI, a proxy for fatty liver) were measured. It is assumed that a further understanding of the relationships between the biomarker levels and various determinants of health may improve our possibilities for developing novel predictive algorithms for use in interventions aimed at reducing morbidity associated with modifiable risk factors of lifestyle.

## 2. Materials and Methods

### 2.1. Study Design, Data Sources and Participants

Data were collected from a cross-sectional population-based health survey (the National FINRISK Study) carried out in Finland in 2002. In this survey, an age- and gender-stratified random sample was drawn from the population register according to an international protocol [16]. The assessments included detailed records on pre-existing medical conditions, physical and anthropometric measures, laboratory tests and detailed structured information on alcohol use, smoking, coffee consumption and physical activity using questionnaires, which have been previously validated for international population-based health studies [16,17,18]. Data was available from 8743 participants (4048 men, 4695 women) (mean age 48 ± 13 years, range 25–74 years), who completed the questionnaires and attended the medical examinations and blood sampling for laboratory tests. 

Body weight and height were measured to the nearest 0.1 kg and 0.1 cm, respectively. Body mass index (BMI) (kg/m^2^) was calculated as an index of relative body weight. Waist circumference was determined to the nearest 0.5 cm between the lowest rib and the iliac crest while exhaling. 

Information on alcohol consumption from the past 12 months prior to blood sampling were recorded using questionnaires covering the total amounts of ethanol-containing drinks, frequencies of consumption and the types of beverages consumed as previously described [19]. The amount of ethanol in different beverages was quantitated in grams of ethanol and expressed as defined portion sizes (standard drink corresponding to 12 g of alcohol). The data on smoking habits and coffee consumption were expressed as the amounts of cigarettes per day and the intake of standard servings of coffee (cups) per day, respectively. Leisure-time physical activity and the number of physical exercises with intensity leading to shortness of breath or sweating were registered as previously described [13]. 

The data on various lifestyle risk factors was subsequently used to define low risk (=0), medium risk (=1) and high risk (=2) categories for each individual parameter, as follows: alcohol consumption, 0 = no consumption (abstainers); 1 = moderate drinking: alcohol consumption between 1–14 (men) or 1–7 (women) standard drinks per week; 2 = heavy drinking, alcohol consumption exceeds 14 drinks (men) or 7 drinks (women) per week; smoking, 0 = no smoking, 1 = 1–19 cigarettes per day, 2 = ≥20 cigarettes per day; BMI, 0 = <25; 1 = ≥25 and <30; 2 = ≥30; physical activity, 0 = over 4 h per week; 1 = 0.5 and 4 h per week and 2 = less than 30 min per week. In addition, the total sum of the above scores was calculated for each individual to provide total lifestyle risk factor scores (LRFS) with higher scores (maximum = 8) indicating an unhealthier lifestyle [7,13].

The data on medical examinations and health records were used to classify the subjects according to their pre-existing medical conditions, which included the following partially overlapping conditions: fatty liver (*n* = 2681; 30.7%), hypertension (*n* = 1681; 19.2%), diabetes or abnormal oral glucose test (*n* = 422; 4.8%), coronary heart disease (*n* = 210; 2.4%), cerebrovascular diseases (*n* = 171; 2.0%), angina pectoris (*n* = 331; 3.8%), cardiac insufficiency (*n* = 221; 2.5%), malignancy (*n* = 97; 1.1%), asthma (*n* = 430; 4.9%), chronic bronchitis (*n* = 214; 2.4%), gallbladder disease (*n* = 115; 1.3%), rheumatic arthritis (*n* = 115; 1.3%), joint disorders (*n* = 777; 8.9%), degenerative back pain (*n* = 1411; 16.1%), kidney or urinary tract diseases (*n* = 170; 1.9%), depression (*n* = 624; 7.2%) and other psychiatric disorders (*n* = 165; 1.9%). The material did not include hospitalized patients, individuals with severe liver diseases or active infections at the time of blood sampling. 

The approval for the study was received from the Coordinating Ethics Committee of the Helsinki and Uusimaa Hospital District and from the Ethics Committee of the National Public Health Institute (2002:87/2001). All surveys were conducted in accordance with the Declaration of Helsinki according to the ethical rules of the National Public Health Institute. 

### 2.2. Laboratory Analyses

Serum liver enzymes (ALT and GGT) were measured by standard clinical chemical methods on an Abbott Architect clinical chemistry analyzer following the recommendations of the assay manufacturer (Abbott Laboratories, Abbott Park, IL, USA). High-sensitivity CRP, a biomarker of inflammation, was determined using a latex immunoassay (Sentinel Diagnostics, Milan, Italy) with the Abbott Architect c8000 clinical chemistry analyzer. Lipid profiles included determinations of total cholesterol, high-density lipoprotein-associated cholesterol (HDL), low-density lipoprotein (LDL) and total triglycerides using standard enzymatic methods. The cut-offs for the normal limits of the different biomarkers were as follows: ALT (50 U/L men; 35 U/L women), GGT (60 U/L men; 40 U/L women), CRP (3.0 mg/L), cholesterol (5 mmol/L), HDL cholesterol (1.0 mmol/L men, 1.2 mmol/L women), LDL cholesterol (3.0 mmol/L), triglycerides (1.7 mmol/L). Fatty liver index, a predictor algorithm for fatty liver disease, was analyzed based on BMI, waist circumference, triglycerides and GGT, as previously described [13,20].

### 2.3. Statistical Methods

Continuous variables are reported as means and standard deviations (SDs) and compared between groups using Student’s *t*-test. Categorical variables are presented as frequencies and percentages and compared using a chi-square test or Fisher’s exact test, as appropriate. For ordered categorical variables, chi-square test for trend was applied. Binary logistic regression analysis was applied to evaluate the associations between pre-existing medical conditions and liver enzymes. In further assessment on the associations between distinct disease subgroups and biomarker status, the participants were divided into four groups according to biomarker (negative/positive) and disease status (negative/positive) and a multinomial logistic regression analysis was applied. In all regression analyses BMI, alcohol drinking, smoking status, coffee consumption and physical activity were used as covariates. Results are presented as odds ratios (OR) and 95% confidence intervals (CI). Correlations were calculated using Spearman rank correlation coefficients. For the analyses, SPSS Statistics 28.0 (Armonk, NY, USA: IBM Corp.) software was used. A two-sided *p*-value < 0.05 was considered statistically significant.

## 3. Results

The main demographic and lifestyle characteristics, as divided according to the liver enzyme status, are summarized in Table 1. In this population-based sample, ALT activities exceeded the upper normal limits in 907 (10.4%) individuals (12.1% of men and 8.9% of women in the total population). Abnormal GGT occurred in 1238 (14.2%) of the subjects (17.1% of men and 11.7% of women). Increased activities were more common in men (*p* < 0.0005 for both ALT and GGT) (Table 1). Increased GGT was relatively more prevalent in those over 40 years of age (*p* < 0.001) whereas ALT activities frequently exceeded the upper normal limits also in men younger than 40 years (Table 1). 

Increased prevalence of abnormal ALT and GGT activities were observed in individuals consuming alcohol (ALT: *p* < 0.0005 for men; GGT: *p* < 0.0005 for both men and women), and in those with overweight, most strikingly in the individuals with the most severe degrees of adiposity (*p* < 0.0005 for trend in all comparisons) (Table 1). Smoking (*p* < 0.0005 for GGT in men, *p* = 0.002 in women) and physical inactivity (ALT: *p* < 0.0005 for men, *p* < 0.05 for women; GGT: *p* < 0.0005 for both genders) also showed significant associations with the liver enzyme status. Coffee consumption was also found to be associated with the status of liver enzymes: ALT (*p* < 0.001 for men), GGT (*p* < 0.0005 for both genders) (Table 1). Quantities exceeding three cups per day were associated with relatively lower odds for elevated liver enzymes than those in individuals with low levels of coffee intake. 

The data on ALT and GGT status in relation to various pre-existing medical conditions for all subjects and for women and men separately are summarized in Table 2. An elevated FLI index indicating fatty liver was found in 2681 individuals (30.7% of the total population). Increased blood pressure (hypertension) was recorded in 19.2% of the subjects. Other morbidities observed in this material were diabetes or abnormal oral glucose test (4.8%), coronary heart disease (2.4%), cerebrovascular diseases (2.0%), angina pectoris (3.8%), cardiac insufficiency (2.5%), malignancies (1.1%), asthma (4.9%), chronic bronchitis (2.4%), gallbladder disease (1.3%), rheumatic arthritis (1.3%), joint disorders (8.9%), degenerative back pain (16.1%), kidney or urinary tract diseases (1.9%), depression (7.2%) and other psychiatric disorders (1.9%). Increased serum ALT activities were found to be overrepresented in those with fatty liver (*p* < 0.0005), diabetes (*p* < 0.0005), hypertension (*p* < 0.0005) and psychiatric morbidities (*p* < 0.0005). Elevated GGT activities were also more common in those with fatty liver (*p* < 0.0005), hypertension (*p* < 0.0005) and diabetes (*p* < 0.0005). In addition, abnormal GGT levels were prevalent in individuals with coronary heart disease (*p* < 0.0005), cerebrovascular disease (*p* < 0.01), angina pectoris (*p* < 0.0005), cardiac insufficiency (*p* < 0.0005), asthma (*p* < 0.0005), chronic bronchitis (*p* = 0.001), gallbladder disease (*p* < 0.01), joint disorders (*p* < 0.0005), degenerative back pain (*p* < 0.0005), depression (*p* < 0.0005) and other psychiatric morbidities (*p* < 0.0005) (Table 2). 

Significantly higher lifestyle risk factor scores (LRFS) characterized all subgroups with pre-existing medical conditions when compared with those having no morbidities, the highest scores being found in the individuals with abnormal fatty liver index (Table 3). The total burden of unfavorable lifestyle risk factors was also found to significantly influence the status of liver enzymes (Table 4). Even in subjects with no morbidities the occurrence of abnormal GGT findings was found to increase in a rather linear manner as a function of LRFS (*p* = 0.003 for trend). In the subjects with various pre-existing medical conditions significant dose–response relationships between the actual number of unfavorable lifestyle factors and serum ALT and GGT were noted in several subgroups (Table 4). In these, high LRFS together with liver enzyme abnormalities were strikingly more common than in the corresponding comparisons with the subgroup of individuals with no morbidities. In correlation analyses, the combined sum of lifestyle risk factor scores (LRFS) showed significant correlations with fatty liver index (*r_s_* = 0.542), the activities of liver enzymes (ALT: *r_s_* = 0.252; GGT: *r_s_* = 0.377), indices of inflammation (CRP: *r_s_* = 0.296) and lipid status (cholesterol: *r_s_* = 0.140; HDL-cholesterol: *r_s_* = –0.192; LDL-cholesterol: *r_s_* = 0.153; triglycerides: *r_s_* = 0.289) (*p* < 0.001 for all comparisons).

In logistic regression analysis of the various pre-existing medical conditions as dependent variables as adjusted for alcohol use, smoking, BMI, physical activity and coffee consumption, a significant association was observed with liver enzyme status and fatty liver (ALT: OR 4.8 (3.9–5.9), *p* < 0.0005; GGT: OR 9.6 (7.9–11.8), *p* < 0.0005), diabetes (ALT: OR 1.4 (1.1–1.9), *p* < 0.01; GGT: OR 1.8 (1.4–2.3), *p* < 0.0005), coronary heart disease (GGT: OR 1.9 (1.3–2.6), *p* < 0.001), hypertension (GGT: OR 1.6 (1.4–1.9), *p* < 0.0005), cardiac insufficiency (GGT: OR 1.6 (1.1–2.2), *p* < 0.01), asthma (GGT: OR 1.5 (1.2–1.9), *p* < 0.01), depression (GGT: OR 1.5 (1.2–1.9), *p* < 0.0005) and other psychiatric disorders (GGT: OR 1.7 (1.2–2.5), *p* = 0.006).

Both ALT and GGT activities were found to be prevalent in the individuals with hypertension, which is a condition known to be significantly influenced by lifestyle choices. Further analyses of the biomarker data in this subgroup showed that the odds for hypertension are significantly increased in those with elevated liver enzymes: OR 1.7 (1.3–2.1) if both ALT and GGT were elevated (*p* < 0.0005), 1.3 (1.1–1.5) if only one of these was elevated (*p* = 0.001). Multinomial regression analysis of the biomarker profiles in the individuals with or without hypertension and with or without abnormal ALT activities are shown in Table 5. Hypertensive individuals with high ALT typically also present with high GGT, elevated triglycerides and abnormal FLI. 

## 4. Discussion

The present data derived from a large cross-sectional population-based sample indicate that liver enzyme abnormalities, which have been recognized as an increasingly common phenomenon in current health care, coincide with the burden of modifiable risk factors of lifestyle and simultaneously also characterize a wide array of pre-existing medical conditions. The liver markers also correlate with biomarkers of inflammation, lipid status and fatty liver index suggesting that such biomarkers could probably be used in medical algorithms for more comprehensive health assessment protocols among individuals presenting with unfavorable lifestyle factors [3,5,6,13,21].

The need for predictive algorithms for defining clinically relevant and mechanistically anchored disease subgroups for which optimal treatment strategies can be applied has recently been widely acknowledged [22]. While serum GGT and ALT activities have traditionally been used as tests for screening liver dysfunction, current data suggests the usefulness of following liver enzyme activities also as indicators of adverse metabolic consequences of an unhealthy lifestyle associated with multiple health problems. The findings also support a close interplay between hepatic and extrahepatic conditions and emphasizes the importance of simultaneous management of such multi-morbidity [23].

Current findings are consistent with previous observations showing elevated liver enzymes in patients with diabetes and cardiovascular morbidity [2,3,4,5,24,25,26,27]. GGT activities were, however, found here to associate with a striking number of additional heterogeneous extrahepatic disease entities. While previous studies have reported significant links between GGT and cardiovascular diseases, diabetes, metabolic syndrome, cancer, neurodegenerative conditions and rheumatic diseases [1,5,24,28,29,30], the present findings further indicate significant associations between GGT, lifestyle and conditions such as adult asthma, degenerative back pain, joint disorders and psychiatric morbidities. These findings support the view that lifestyle risk factors may play a pivotal role behind such conditions [31,32]. Changes in GGT activities have also previously been suggested to predict cardio- and cerebrovascular mortality and disability pensions [5,33]. The most striking cardiovascular risks may occur in those who simultaneously present with hepatic steatosis [34]. The development of atherosclerosis and fatty liver may also be mechanistically linked with each other through GGT as an inducer of iron-dependent LDL oxidation [35]. 

The present multinomial logistic regression data, adjusted for the various lifestyle risk factors, revealed a strong association between increased liver enzyme activities and hypertension, particularly in those with increased activities in both ALT and GGT. Hypertension, known to be significantly influenced by lifestyle, is currently the leading preventable cardiovascular risk factor affecting approximately 30% of the adult population in Western countries [36]. Both hypertension and fatty liver are characterized by the absence of warning signs or symptoms, highlighting the need for improvement in their early-phase detection. Fatty liver observed here in one third of the population may represent the hepatic manifestation of the metabolic syndrome and constitute a major cause of unexpected liver enzyme abnormalities in general populations especially in individuals with obesity [23,37,38,39,40,41,42]. On the other hand, fatty liver can drive hypertension through the development of insulin resistance, dyslipidemia, oxidative stress, and systemic inflammation [21,23,27,30,39,43,44,45]. Obesity-associated hypertension is characterized by systemic vascular resistance and arterial stiffness and seems to represent a distinct clinical phenotype accounting for up to two-thirds of the risk for human essential hypertension [43,44,45,46,47]. 

The current data show that the lifestyle-associated metabolic burden is significantly increased as a function of the total number of unfavorable lifestyle factors even in individuals without any apparent pre-existing medical conditions, suggesting that the biomarker responses represent early changes in the sequence of events leading from risk exposure to disease outcomes. Interestingly, in individuals with pre-existing medical conditions the LFRS levels were systematically higher, suggesting that the likelihood for such conditions is also significantly driven by lifestyle. From a public health perspective, these findings also underscore the importance of interventions aimed at reducing the total number of lifestyle risk factors [7,11,13,15,48,49,50,51,52].

The main individual determinants of a healthy lifestyle include alcohol drinking in moderation, weight control, not smoking and taking regular exercise. In addition to liver function abnormalities, typical pathophysiological consequences created by unfavorable factors of lifestyle include an abnormal status of inflammation, oxidative stress and altered fatty acid metabolism [50,53]. Accordingly, current data shows that the lipid profiles and inflammatory status correlate with the activities of liver enzymes, fatty liver index and the total burden of lifestyle risk factors. Not surprisingly, biomarkers of inflammation have been shown to yield predictive value in assessing cardiovascular morbidity even in individuals without apparent atherosclerotic manifestations [54,55].

Among the various individual lifestyle risk factors, alcohol drinking has been established as a major contributor to both hepatic and multiple extrahepatic health risks, including cardiac insufficiency [56,57], adverse brain outcomes [58,59], carcinogenesis [60,61,62] and all-cause mortality [63,64]. In liver tissue, alcohol use and obesity induce similar histological manifestations and pathogenic pathways, such as cytochrome P450 enzyme activation and oxidative stress, thereby exacerbating toxicity in a supra-additive manner [65,66,67,68,69,70,71,72,73,74,75]. This data should also be considered in the calibration of thresholds of alcohol intake levels used to differentiate between non-alcoholic and alcoholic causes of fatty liver disease [76,77].

Synergistic interactions also occur between the frequently co-occurring habits of alcohol use and smoking [77,78,79,80,81,82], which seems to be a potent effect modifier in alcohol-induced GGT enzyme induction and the metabolism of extracellular glutathione [83]. Changes in GGT may reflect the status of oxidative stress and the body’s need to maintain intracellular GSH in response to the metabolic burden created by unfavorable lifestyle factors [73,74,75,84]. On the other hand, coffee consumption has been suggested to provide protection towards the likelihood for abnormal liver enzyme status [85,86]. Coffee is a rich source of antioxidants and coffee drinkers have been previously shown to exhibit relatively lower liver enzyme activities both in general populations and among alcohol consumers [85,86]. However, the effects may depend on the quantities of coffee consumed since the alleviating effects seem to be restricted to those who consume at least four cups of coffee per day [85,87,88]. 

Physical activity appears to be an effective modulator of the status of liver enzyme activities. Regular physical exercise is known to lead to more favorable metabolic profiles and reduction in the levels of the biomarkers of inflammation and liver status [50,89,90,91,92,93,94,95]. Physical activity may also play a role in regulating the status of inflammation and oxidative stress [35,50,96,97]. In obese individuals, liver enzyme activities have been shown to correlate with the degree of fat deposition and to decline with losing weight. Furthermore, moderate to vigorous physical activity may decrease the amount of fat in liver tissue even in the absence of weight loss [50,98,99]. A recent study based on the UK biobank data further found that physically active individuals have longer life expectancies across all levels of overweight when compared with those with sedentary activity [94]. 

The strengths of this study include a large number of subjects and a comprehensive assessment of the characteristics of lifestyle determinants and a wide array of pre-existing medical conditions together with measurements of several biomarkers reflecting liver function, inflammation and lipid status. The study also included separate assessments for women and men. Nevertheless, there are potential limitations to consider. Due to the observational and cross-sectional nature of the study and lack of follow-up data it is not possible to establish causal associations. The data on the determinants of lifestyle were based on self-reports which may cause underreporting particularly in variables reflecting less socially desirable behaviors, such as alcohol consumption. However, this is more likely to dilute our findings than overestimate the observed associations. 

Taken together, our study demonstrates previously unrecognized relationships between liver enzymes, factors of lifestyle and human diseases. The observed associations between liver status and various extrahepatic conditions, such as hypertension, may also have important implications for public health policies. The data also underscores the potential for developing biomarker-based algorithms to provide predictive information for interventions targeting health risks related to unfavorable factors of lifestyle. 

## Figures and Tables

**Table 1:** Main demographic characteristics of the study material, as classified according to lifestyle factors and liver enzyme status.

		All, *n* = 8613–8743					Men, *n* = 3989–4048					Women, *n* = 4624–4695				
		*n*	ALT Elevated	*p*	GGT Elevated	*p*	*n*	ALT Elevated	*p*	GGT Elevated	*p*	*n*	ALT Elevated	*p*	GGT Elevated	*p*
Sex, %	men	4048	490 (12.1)	<0.0005	691 (17.1)	<0.0005										
	women	4695	417 (8.9)		547 (11.7)											
Age	≤40 years	2797	289 (10.3)	0.930	239 (8.5)	<0.0005	1202	178 (14.8)	0.001	154 (12.8)	<0.0005	1595	111 (7.0)	0.001	85 (5.3)	0.001
	>40 years	5946	618 (10.4)		999 (16.8)		2846	312 (11.0)		537 (18.9)		3100	306 (9.9)		462 (14.9)	
Alcohol, drinks/week	abstainers	2994	258 (8.6)	<0.0005	326 (10.9)	<0.0005	1070	100 (9.3)	<0.0005	124 (11.6)	<0.0005	1924	158 (8.2)	0.056	202 (10.5)	<0.0005
	≤14/≤7	4408	438 (9.9)		563 (12.8)		2225	245 (11.0)		335 (15.1)		2183	193 (8.8)		228 (10.4)	
	>14/>7	1256	203 (16.2)		337 (26.8)		709	140 (19.7)		222 (31.3)		547	63 (11.5)		115 (21.0)	
BMI, kg/m^2^	<18.5	62	1 (1.6)	<0.0005	5 (8.1)	<0.0005	13	1 (7.7)	<0.0005	2 (15.4)	<0.0005	49	0 (0.0)	<0.0005	3 (6.1)	<0.0005
	18.5−24.99	3275	158 (4.8)		230 (7.0)		1215	45 (3.7)		96 (7.9)		2060	113 (5.5)		134 (6.5)	
	25−29.99	3510	359 (10.2)		507 (14.4)		1929	220 (11.4)		322 (16.7)		1581	139 (8.8)		185 (11.7)	
	30−34.99	1379	267 (19.4)		340 (24.7)		700	159 (22.7)		202 (28.9)		679	108 (15.9)		138 (20.3)	
	35−39.99	406	86 (21.2)		115 (28.3)		155	46 (29.7)		52 (33.5)		251	40 (15.9)		63 (25.1)	
	≥40.0	111	36 (32.4)		41 (36.9)		36	19 (52.8)		17 (47.2)		75	17 (22.7)		24 (32.0)	
Waist, cm	<94/<80	3870	204 (5.3)	<0.0005	274 (7.1)	<0.0005	1882	106 (5.6)	<0.0005	169 (9.0)	<0.0005	1988	98 (4.9)	<0.0005	105 (5.3)	<0.0005
	94−102/80−88	2294	237 (10.3)		343 (15.0)		1129	151 (13.4)		218 (19.3)		1165	86 (7.4)		125 (10.7)	
	>102/>88	2529	463 (18.3)		620 (24.5)		1033	232 (22.5)		303 (29.3)		1496	231 (15.4)		317 (21.2)	
Smoking	none	6338	650 (10.3)	0.094	806 (12.7)	<0.0005	2696	320 (11.9)	0.398	410 (15.2)	<0.0005	3642	330 (9.1)	0.479	396 (10.9)	0.002
	1−19 cigarettes/day	1575	152 (9.7)		237 (15.0)		737	87 (11.8)		123 (16.7)		838	65 (7.8)		114 (13.6)	
	≥20 cigarettes/day	759	95 (12.5)		186 (24.5)		570	79 (13.9)		152 (26.7)		189	16 (8.5)		34 (18.0)	
Physical activity	>4 h/week	1984	149 (7.5)	<0.0005	159 (8.0)	<0.0005	1016	83 (8.2)	<0.0005	97 (9.5)	<0.0005	968	66 (6.8)	0.029	62 (6.4)	<0.0005
	0.5−4 h/week	4661	474 (10.2)		693 (14.9)		2068	238 (11.5)		373 (18.0)		2593	236 (9.1)		320 (12.3)	
	<0.5 h/week	1968	268 (13.6)		368 (18.7)		905	161 (17.8)		213 (23.5)		1063	107 (10.1)		155 (14.6)	
Coffee, cups/day	none	951	97 (10.2)	0.002	106 (11.1)	<0.0005	373	46 (12.3)	0.001	56 (15.0)	<0.0005	578	51 (8.8)	0.054	50 (8.7)	<0.0005
	1−3 cups/day	2817	339 (12.0)		484 (17.2)		1065	161 (15.1)		227 (21.3)		1752	178 (10.2)		257 (14.7)	
	≥4 cups/day	4957	470 (9.5)		643 (13.0)		2603	282 (10.8)		405 (15.6)		2354	188 (8.0)		238 (10.1)	

**Table 2:** Numbers and frequencies (%) of abnormal ALT and GGT levels in the study population with and without various pre-existing medical conditions.

		All, *n* = 8591–8743					Men, *n* = 3974–4048					Women, *n* = 4617–4695				
		*n*	ALT Elevated	*p*	GGT Elevated	*p*	*n*	ALT Elevated	*p*	GGT Elevated	*p*	*n*	ALT Elevated	*p*	GGT Elevated	*p*
Morbidities	no	3784	181 (4.8)	<0.0005	177 (4.7)	<0.0005	1459	60 (4.1)	<0.0005	61 (4.2)	<0.0005	2325	121 (5.2)	<0.0005	116 (5.0)	<0.0005
	yes	4959	726 (14.6)		1061 (21.4)		2589	430 (16.6)		630 (24.3)		2370	296 (12.5)		431 (18.2)	
Fatty liver (FLI ≥ 60)	no	6012	300 (5.0)	<0.0005	358 (6.0)	<0.0005	2282	87 (3.8)	<0.0005	118 (5.2)	<0.0005	3730	213 (5.7)	<0.0005	240 (6.4)	<0.0005
	yes	2681	604 (22.5)		879 (32.8)		1762	402 (22.8)		572 (32.5)		919	202 (22.0)		307 (33.4)	
Diabetes/abnormal	no	8169	811 (9.9)	<0.0005	1094 (13.4)	<0.0005	3763	446 (11.9)	0.014	614 (16.3)	<0.0005	4406	365 (8.3)	<0.0005	480 (10.9)	<0.0005
OGT	yes	422	80 (19.0)		112 (26.5)		211	37 (17.5)		63 (29.9)		211	43 (20.4)		49 (23.2)	
Coronary heart disease	no	8478	876 (10.3)	0.460	1172 (13.8)	<0.0005	3849	469 (12.2)	0.324	642 (16.7)	0.022	4629	407 (8.8)	0.014	530 (11.4)	<0.0005
	yes	210	80 (19.0)		52 (24.8)		166	16 (9.6)		39 (23.5)		44	9 (20.5)		13 (29.5)	
Cerebrovascular disease	no	8518	887 (10.4)	0.486	1190 (14.0)	0.004	3918	477 (12.2)	0.469	663 (16.9)	0.331	4600	410 (8.9)	0.629	527 (11.5)	0.003
	yes	171	15 (8.8)		37 (21.6)		102	10 (9.8)		21 (20.6)		69	5 (7.2)		16 (23.2)	
Hypertension	no	6992	651 (9.3)	<0.0005	834 (11.9)	<0.0005	3193	355 (11.1)	<0.0005	471 (14.8)	<0.0005	3799	296 (7.8)	<0.0005	363 (9.6)	<0.0005
	yes	1681	248 (14.8)		388 (23.1)		821	130 (15.8)		211 (25.7)		860	118 (13.7)		177 (20.6)	
Cardiac insufficiency	no	8456	883 (10.4)	0.186	1170 (13.8)	<0.0005	3874	475 (12.3)	0.044	643 (16.6)	0.001	4582	408 (8.9)	0.844	527 (11.5)	0.033
	yes	221	17 (7.7)		54 (24.4)		137	9 (6.6)		38 (27.7)		84	8 (9.5)		16 (19.0)	
Angina pectoris	no	8356	872 (10.4)	0.327	1153 (13.8)	<0.0005	3811	470 (12.3)	0.032	634 (16.6)	0.007	4545	402 (8.8)	0.378	519 (11.4)	0.004
	yes	331	29 (8.8)		74 (22.4)		205	15 (7.3)		49 (23.9)		126	14 (11.1)		25 (19.8)	
Malignancy	no	8589	893 (10.4)	0.307	1209 (14.1)	0.696	3969	483 (12.2)	0.036	674 (17.0)	0.705	4620	410 (8.9)	0.448	535 (11.6)	0.332
	yes	97	7 (7.2)		15 (15.5)		47	1 (2.1)		7 (14.9)		50	6 (12.0)		8 (16.0)	
Asthma	no	8247	844 (10.2)	0.123	1132 (13.7)	<0.0005	3860	457 (11.8)	0.087	643 (16.7)	0.007	4387	387 (8.8)	0.361	489 (11.1)	<0.0005
	yes	430	54 (12.6)		91 (21.2)		152	25 (16.4)		38 (25.0)		278	29 (10.4)		53 (19.1)	
Chronic bronchitis	no	8469	875 (10.3)	0.522	1176 (13.9)	0.001	3904	473 (12.1)	0.522	656 (16.8)	0.152	4565	402 (8.8)	0.107	520 (11.4)	0.001
	yes	214	25 (11.7)		47 (22.0)		109	11 (10.1)		24 (22.0)		105	14 (13.3)		23 (21.9)	
Gallbladder disease	no	8573	889 (10.4)	0.558	1197 (14.0)	0.008	3978	477 (12.0)	0.393	671 (16.9)	0.075	4595	412 (9.0)	0.134	526 (11.4)	0.022
	yes	115	10 (8.7)		26 (22.6)		40	7 (17.5)		11 (27.5)		75	3 (4.0)		15 (20.0)	
Rheumatic arthritis	no	8568	890 (10.4)	0.774	1203 (14.0)	0.119	3985	484 (12.1)	0.173	675 (16.9)	0.515	4583	406 (8.9)	0.293	528 (11.5)	0.058
	yes	115	11 (9.6)		22 (19.1)		33	1 (3.0)		7 (21.2)		82	10 (12.2)		15 (18.3)	
Joint disorders	no	7883	814 (10.3)	0.593	1070 (13.6)	<0.0005	3686	457 (12.4)	0.036	613 (16.6)	0.052	4197	357 (8.5)	0.003	457 (10.9)	<0.0005
	yes	777	85 (10.9)		152 (19.6)		321	27 (8.4)		67 (20.9)		456	58 (12.7)		85 (18.6)	
Degenerative back pain	yes	7250	735 (10.1)	0.130	977 (13.5)	<0.0005	3309	405 (12.2)	0.447	539 (16.3)	0.009	3941	330 (8.4)	0.004	438 (11.1)	0.027
	no	1411	162 (11.5)		242 (17.2)		696	78 (11.2)		142 (20.4)		715	84 (11.7)		100 (14.0)	
Kidney or urinary	yes	8507	883 (10.4)	0.930	1196 (14.1)	0.371	3954	478 (12.1)	0.965	669 (16.9)	0.240	4553	405 (8.9)	0.757	527 (11.6)	0.578
tract diseases	no	170	18 (10.6)		28 (16.5)		57	7 (12.3)		13 (22.8)		113	11 (9.7)		15 (13.3)	
Depression	yes	8054	823 (10.2)	0.094	1089 (13.5)	<0.0005	3763	449 (11.9)	0.331	613 (16.3)	<0.0005	4291	374 (8.7)	0.102	476 (11.1)	<0.0005
	no	624	77 (12.3)		136 (21.8)		250	35 (14.0)		68 (27.2)		374	42 (11.2)		68 (18.2)	
Other psychiatric	yes	8509	871 (10.2)	0.005	1177 (13.8)	<0.0005	3934	469 (11.9)	0.024	654 (16.6)	<0.0005	4575	402 (8.8)	0.095	523 (11.4)	0.006
disorders	no	165	28 (17.0)		46 (27.9)		79	16 (20.3)		28 (35.4)		86	12 (14.0)		18 (20.9)	

**Table 3:** Lifestyle risk factor scores (LRFS) (mean, SD) in individuals with or without pre-existing medical conditions. *p* values indicate the difference for comparisons with individuals with no morbidities.

Pre-Existing Condition		LRFS	
	*n*	Mean (sd)	*p*
No morbidities	3690	2.5 (1.4)	
FLI ≥ 60 (fatty liver)	2586	4.0 (1.3)	<0.0005
Coronary heart disease	191	3.4 (1.3)	<0.0005
Cerebrovascular disease	159	3.3 (1.3)	<0.0005
Hypertension	1627	3.4 (1.4)	<0.0005
Cardiac insufficiency	203	3.4 (1.3)	<0.0005
Angina pectoris	309	3.3 (1.4)	<0.0005
Malignancy	92	3.0 (1.3)	<0.0005
Asthma	419	3.2 (1.4)	<0.0005
Chronic bronchitis	204	3.6 (1.5)	<0.0005
Gallbladder disease	112	3.3 (1.4)	<0.0005
Rheumatic arthritis	111	3.2 (1.4)	<0.0005
Joint disorders	758	3.3 (1.5)	<0.0005
Degenerative back pain	1361	3.2 (1.4)	<0.0005
Kidney or urinary tract disease	157	3.1 (1.5)	<0.0005
Depression	608	3.4 (1.6)	<0.0005
Other psychiatric disorders	154	3.8 (1.5)	<0.0005

**Table 4:** ALT and GGT enzyme status in the study material classified according to the number of lifestyle risk factors (LRFS) and the presence or absence of pre-existing clinical conditions.

		LRFS 0–1	LRFS 2–3	LRFS 4–5	LRFS 6–8	*p*
No morbidities		*n* = 908	*n* = 1982	*n* = 710	*n* = 90	
	ALT elevated	42 (4.6%)	87 (4.4%)	30 (4.2%)	7 (7.8%)	0.761
	GGT elevated	35 (3.9%)	83 (4.2%)	38 (5.4%)	12 (13.3%)	0.003
Fatty liver (FLI ≥ 60)		*n* = 36	*n* = 942	*n* = 1297	*n* = 311	
	ALT elevated	3 (8.3%)	183 (19.4%)	311 (24.0%)	93 (29.9%)	<0.0005
	GGT elevated	10 (27.8%)	257 (27.3%)	429 (33.1%)	161 (51.8%)	<0.0005
Diabetes/abnormal OGT		*n* = 23	*n* = 181	*n* = 165	*n* = 34	
	ALT elevated	0 (0.0%)	26 (14.4%)	39 (23.6%)	11 (32.4%)	<0.0005
	GGT elevated	1 (4.3%)	40 (22.1%)	48 (29.1%)	20 (58.8%)	<0.0005
Coronary heart disease		*n* = 15	*n* = 88	*n* = 78	*n* = 10	
	ALT elevated	1 (6.7%)	5 (5.7%)	15 (19.2%)	1 (10.0%)	0.056
	GGT elevated	2 (13.3%)	20 (22.7%)	23 (29.5%)	4 (40.0%)	0.082
Cerebrovascular disease		*n* = 15	*n* = 77	*n* = 56	*n* = 11	
	ALT elevated	1 (6.7%)	4 (5.2%)	5 (8.9%)	4 (36.4%)	0.025
	GGT elevated	2 (13.3%)	11 (14.3%)	17 (30.4%)	5 (45.5%)	0.005
Hypertension		*n* = 120	*n* = 771	*n* = 631	*n* = 105	
	ALT elevated	2 (1.7%)	82 (10.6%)	130 (20.6%)	27 (25.7%)	<0.0005
	GGT elevated	7 (5.8%)	134 (17.4%)	186 (29.5%)	54 (51.4%)	<0.0005
Cardiac insufficiency		*n* = 20	*n* = 83	*n* = 92	*n* = 8	
	ALT elevated	0 (0.0%)	4 (4.8%)	13 (14.1%)	0 (0.0%)	0.054
	GGT elevated	2 (10.0%)	17 (20.5%)	29 (31.5%)	3 (37.5%)	0.015
Angina pectoris		*n* = 22	*n* = 164	*n* = 101	*n* = 22	
	ALT elevated	2 (9.1%)	9 (5.5%)	11 (10.9%)	3 (13.6%)	0.152
	GGT elevated	1 (4.5%)	27 (16.5%)	31 (30.7%)	10 (45.5%)	<0.0005
Malignancy		*n* = 10	*n* = 51	*n* = 28	*n* = 3	
	ALT elevated	1 (10.0%)	3 (5.9%)	3 (10.7%)	0 (0.0%)	1.000
	GGT elevated	1 (10.0%)	2 (3.9%)	8 (28.6%)	3 (100.0%)	<0.0005
Asthma		*n* = 40	*n* = 211	*n* = 143	*n* = 25	
	ALT elevated	2 (5.0%)	24 (11.4%)	23 (16.1%)	5 (20.0%)	0.026
	GGT elevated	2 (5.0%)	31 (14.7%)	47 (32.9%)	11 (44.0%)	<0.0005
Chronic bronchitis		*n* = 13	*n* = 84	*n* = 86	*n* = 21	
	ALT elevated	0 (0.0%)	8 (9.5%)	12 (14.0%)	5 (23.8%)	0.035
	GGT elevated	2 (15.4%)	15 (17.9%)	23 (26.7%)	6 (28.6%)	0.125
Gallbladder disease		*n* = 10	*n* = 55	*n* = 40	*n* = 7	
	ALT elevated	0 (0.0%)	5 (9.1%)	4 (10.0%)	1 (14.3%)	0.376
	GGT elevated	2 (20.0%)	9 (16.4%)	10 (25.0%)	5 (71.4%)	0.022
Rheumatic arthritis		*n* = 8	*n* = 55	*n* = 42	*n* = 6	
	ALT elevated	0 (0.0%)	2 (3.6%)	9 (21.4%)	0 (0.0%)	0.070
	GGT elevated	0 (0.0%)	9 (16.4%)	11 (26.2%)	1 (16.7%)	0.170
Joint disorders		*n* = 82	*n* = 361	*n* = 262	*n* = 53	
	ALT elevated	2 (2.4%)	34 (9.4%)	39 (14.9%)	9 (17.0%)	<0.0005
	GGT elevated	5 (6.1%)	59 (16.3%)	64 (24.4%)	23 (43.4%)	<0.0005
Degenerative back pain		*n* = 152	*n* = 672	*n* = 457	*n* = 80	
	ALT elevated	4 (2.6%)	68 (10.1%)	67 (14.7%)	20 (25.0%)	<0.0005
	GGT elevated	7 (4.6%)	100 (14.9%)	106 (23.2%)	24 (30.0%)	<0.0005
Kidney or		*n* = 23	*n* = 69	*n* = 57	*n* = 8	
urinary tract disease	ALT elevated	2 (8.7%)	5 (7.2%)	11 (19.3%)	0 (0.0%)	0.340
	GGT elevated	2 (8.7%)	7 (10.1%)	14 (24.6%)	5 (62.5%)	<0.0005
Depression		*n* = 66	*n* = 276	*n* = 204	*n* = 62	
	ALT elevated	4 (6.1%)	28 (10.1%)	29 (14.2%)	14 (22.6%)	0.002
	GGT elevated	2 (3.0%)	47 (17.0%)	56 (27.5%)	29 (46.8%)	<0.0005
Other psychiatric disorders		*n* = 7	*n* = 61	*n* = 63	*n* = 23	
	ALT elevated	1 (14.3%)	9 (14.8%)	8 (12.7%)	8 (34.8%)	0.132
	GGT elevated	1 (14.3%)	13 (21.3%)	17 (27.0%)	11 (47.8%)	0.019

**Table 5:** Odds ratios for biomarker profiles in groups classified according to ALT (normal/elevated) status and the presence or absence of hypertension (HT+/HT−), as adjusted for various factors (alcohol, smoking, BMI, coffee and physical activity) (multinomial regression analysis).

All	ALT− HT+		ALT+ HT−		ALT+ HT+	
	*n* = 1379–1383	*p*	*n* = 628–630	*p*	*n* = 240–241	*p*
GGT normal	1.0		1.0		1.0	
GGT elevated	1.7 (1.4–2.0)	<0.0005	7.5 (6.2–9.0)	<0.0005	12.2 (9.1–16.3)	<0.0005
Cholesterol normal	1.0		1.0		1.0	
Cholesterol elevated	1.2 (1.0–1.3)	0.031	1.3 (1.0–1.5)	0.019	1.3 (0.9–1.7)	0.142
HDL normal	1.0		1.0		1.0	
HDL decreased	1.2 (1.0–1.5)	0.013	1.9 (1.5–2.3)	<0.0005	1.5 (1.0–2.0)	0.025
LDL normal	1.0		1.0		1.0	
LDL elevated	1.0 (0.9–1.2)	0.499	1.4 (1.1–1.7)	0.001	1.2 (0.9–1.6)	0.270
Triglycerides normal	1.0		1.0		1.0	
Triglycerides elevated	1.7 (1.5–1.9)	<0.0005	2.1 (1.7–2.5)	<0.0005	2.9 (2.2–3.8)	<0.0005
hs-CRP normal	1.0		1.0		1.0	
hs-CRP elevated	1.3 (1.2–1.6)	<0.0005	1.1 (0.9–1.3)	0.429	1.4 (1.1–1.9)	0.013
FLI <60	1.0		1.0		1.0	
FLI ≥ 60	2.0 (1.7–2.3)	<0.0005	4.7 (3.7–5.9)	<0.0005	9.2 (5.9–14.3)	<0.0005

## Data Availability

THL Biobank administrates and grants access to the FINRISK data to research projects that are of high scientific quality and impact, are ethically conducted, and that correspond with the research areas of THL Biobank. All data are available for application at https://thl.fi/en/web/thl-biobank/for-researchers/sample-collections/the-national-finrisk-study-1992-2012 (accessed on 26 April 2023). The name of dataset is the National FINRISK Study 1992–2012. Interested researchers can replicate our study findings in their entirety by directly obtaining the data and following the protocol in the Methods section. The authors did not have any special access privileges that others would not have. More information: finriski(at)thl.fi.
